# ILP-based maximum likelihood genome scaffolding

**DOI:** 10.1186/1471-2105-15-S9-S9

**Published:** 2014-09-10

**Authors:** James Lindsay, Hamed Salooti, Ion Măndoiu, Alex Zelikovsky

**Affiliations:** 1 Computer Science and Engineering Department, University of Connecticut, 371 Fairfield Way, Storrs, CT 06269-4155, USA; 2 Department of Computer Science, Georgia State University, 34 Peachtree Street, Atlanta, GA 30303, USA

## Abstract

**Background:**

Interest in *de novo *genome assembly has been renewed in the past decade due to rapid advances in high-throughput sequencing (HTS) technologies which generate relatively short reads resulting in highly fragmented assemblies consisting of contigs. Additional long-range linkage information is typically used to orient, order, and link contigs into larger structures referred to as *scaffolds*. Due to library preparation artifacts and erroneous mapping of reads originating from repeats, scaffolding remains a challenging problem. In this paper, we provide a scalable scaffolding algorithm (SILP2) employing a maximum likelihood model capturing read mapping uncertainty and/or non-uniformity of contig coverage which is solved using integer linear programming. A Non-Serial Dynamic Programming (NSDP) paradigm is applied to render our algorithm useful in the processing of larger mammalian genomes. To compare scaffolding tools, we employ novel quantitative metrics in addition to the extant metrics in the field. We have also expanded the set of experiments to include scaffolding of low-complexity metagenomic samples.

**Results:**

SILP2 achieves better scalability throughg a more efficient NSDP algorithm than previous release of SILP. The results show that SILP2 compares favorably to previous methods OPERA and MIP in both scalability and accuracy for scaffolding single genomes of up to human size, and significantly outperforms them on scaffolding low-complexity metagenomic samples.

**Conclusions:**

Equipped with NSDP, SILP2 is able to scaffold large mammalian genomes, resulting in the longest and most accurate scaffolds. The ILP formulation for the maximum likelihood model is shown to be flexible enough to handle metagenomic samples.

## Introduction

De novo genome assembly is one of the best studied problems in bioinformatics. Interest in the problem has been renewed in the past decade due to rapid advances in high-throughput sequencing (HTS) technologies, which have orders of magnitude higher throughput and lower cost compared to classic Sanger sequencing. Indeed, top-of-the-line instruments from Illumina and Life Technologies are currently able to generate in a single run billions of reads with an aggregate length of hundreds of gigabases, at a cost of mere cents per megabase. However, most HTS technologies generate relatively short reads, significantly increasing the computational difficulty of the assembly problem. Despite much work on improved assembly algorithms for HTS shotgun reads [1-6], de novo assembly remains challenging, often resulting in highly fragmented assemblies, see [7-14] for recent reviews and benchmarking results. For example, the recent Assemblathon 2 community effort to benchmark de novo genome assemblers [7] shows that the performance of evaluated assemblers is highly variable from dataset to dataset and generally degrades with the complexity of the sample.

To increase the utility of such fragmented assemblies, additional long-range linkage information is typically used to orient, order, and link contigs into larger structures referred to as scaffolds. Although long-range linkage information can be generated using a variety of technologies, including Sanger sequencing of both ends of cloned DNA fragments of up to hundreds of kilobases, Pacific Biosciences reads of up to tens of kilobases [15], and optical maps [16], the most common type of data used in scaffolding are HTS read pairs generated from DNA fragments with length ranging between hundreds of bases to tens of kilobases. While HTS read pairs are relatively easy to generate, the linkage information they provide is noisy due to library preparation artifacts and erroneous mapping of reads originating from repeats. The general scaffolding problem is known to be computationally NP-hard when linkage data contains errors [17]. Moreover, the associated contig orientation and contig ordering problems are intractable as well: the orientation problem is equivalent to finding a maximum bipartite subgraph, whereas the ordering problem is similar to the Optimal Linear Arrangement problem, both of which are NP-hard [18]. Due to the intractability of the problem, greedy heuristics have been employed in practical scaffolding methods such as [17,19]. Scaffolding methods such as SOPRA [20] reduce the size of the problem by iteratively removing inconsistent links and contigs, while MIP [21] heuristically partitions the biconnected components of the scaffolding graph when they are too large to scaffold optimally by mixed integer programming. In SLIQ [22], inequalities are derived from the geometry of contigs to predict the orientation and ordering of adjacent contigs. To find a feasible solution with minimum read pair inconsistency, OPERA [23] provides a novel dynamic programming algorithm.

Algorithms based on explicit statistical models are currently gaining popularity in the area of genome assembly [24], with notable advances in the use of maximum likelihood (ML) methods for both contig assembly [25] and assembly evaluation [26]. In this paper we introduce a highly scalable algorithm based on likelihood maximization for the scaffolding problem. The key step in our algorithm is the selection of contig orientations and a set of read pairs consistent with these orientations (and locally consistent with each other) such that the overall likelihood of selected pairs is maximized. As in previous works [25,26], the likelihood model we employ assumes independence of the HTS read pairs. The currently implemented model takes into account read mapping uncertainty due to overlap with annotated contig repeats as well as variations in contig coverage. The model can be easily extended to incorporate sequencing errors and the distribution of insert lengths; currently we only use the latter to eliminate read pairs with highly discordant insert length lower-bounds and to compute ML estimates for the final gap lengths. Likelihood maximization is formulated as an integer linear program (ILP). Unlike MIP [21], our ILP formulation selects contig orientations and a set of locally consistent read pairs but neither explicitly orders the contigs nor fully guarantees global consistency of selected pairs. The latter are achieved by decomposing the set of selected read pairs into linear paths via bipartite matching.

Scalability of our algorithm, referred to as SILP2, is achieved by adopting a non-serial dynamical programming (NSDP) approach [27]. Rather than solving one large ILP, several smaller ILPs can be solved seperately and composed to find the complete and optimal solution. The order in which the smaller ILPs are solved is determined by the 3-connected components of the underlying scaffolding graph, which can be efficiently identified in linear time via the SPQR-tree data structure [28,29].

Compared to the preliminary version of the algorithm published in [30], referred to as SILP1, SILP2 is based on explicit formalization of likelihood maximization as the optimization objective. We present experiments with several likelihood models capturing read mapping uncertainty and/or non-uniformity of contig coverage. SILP2 also achieves higher scalability by using a more efficient NSDP algorithm than SILP1. This greatly reduces the need for heuristics such as the hierarchical scaffolding approach of SILP1, whereby scaffolding is performed by progressively decreasing the minimum bound on the size of read pair bundles. We have also expanded the set of experiments to include scaffolding of low-complexity metagenomic samples. The results show that SILP2 compares favorably to previous methods OPERA and MIP in both scalability and accuracy for scaffolding single genomes of up to human size, and significantly outperforms them on scaffolding low-complexity metagenomic samples.

## Methods

**Figure 1:**
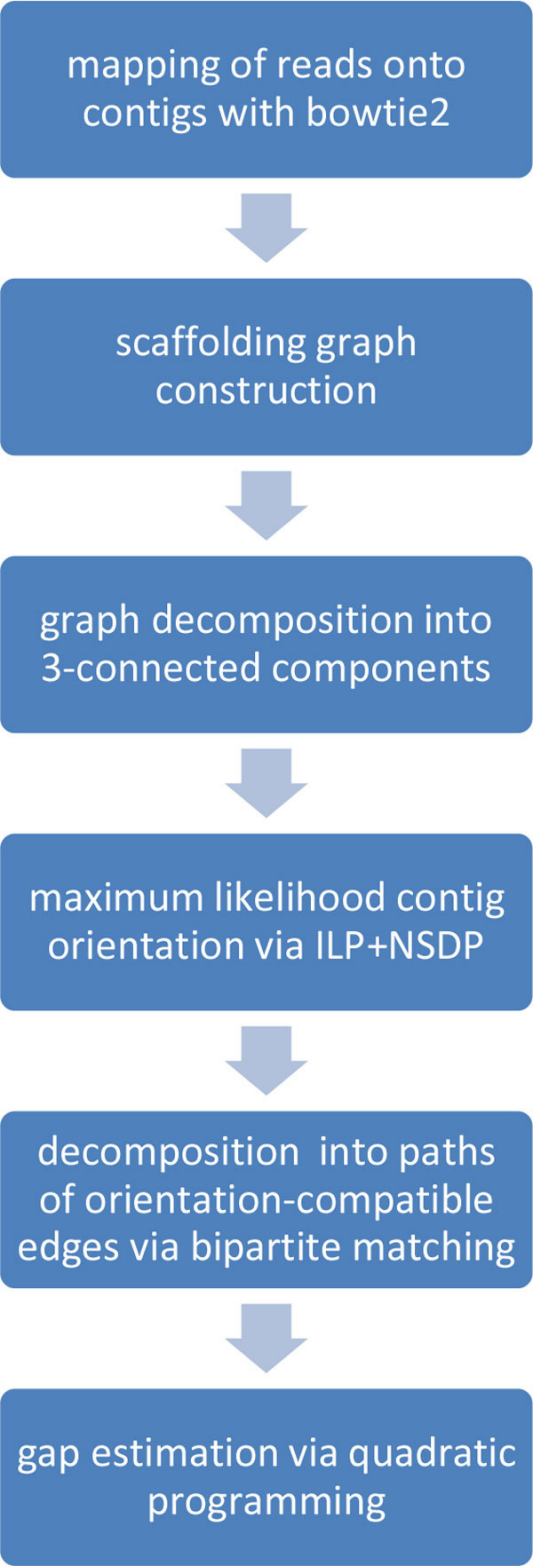
**SILP2 Flowchart**.

Given a set of contigs C and a set of read pairs R, the scaffolding problem asks for the most likely orientation of the contigs along with a partition of the contigs into ordered sets connected by read pairs of R. The main steps of the SILP2 algorithm are as follows (see Figure 1 for a high-level flowchart). We first map the read onto contigs using Bowtie2 [31], disregarding pairing information in the mapping process. Alignments are processed to extract read pairs for which both reads have unique alignments, and the alignments are onto distinct contigs. A scaffolding graph is then constructed with nodes corresponding to contigs and edges corresponding to extracted read pairs. The scaffolding graph is partitioned into 3-connected components using the SPQR tree data structure [28,29] implemented in OGDF [32]. The maximum-likelihood contig orientation is formulated as an ILP that is efficiently solved by applying non-serial dynamic programming based the SPQR tree data structure. Next, scaffold chains are extracted from the ILP solution by using bipartite matching and breaking remaining cycles (see Section S1 in Additional file 1). Finally, maximum likelihood estimates for the gap lengths are obtained using quadratic programming (see Section S2 of Additional file 1). Below we detail the key steps of the algorithm, including scaffolding graph construction, the maximum likelihood models used for contig orientation and mapped read pair probability estimation, then we briefly overview the orientation, the ILP formulation and the improved NSDP algorithm for efficiently solving the ILP.

**Scaffolding graph**. The scaffolding problem is modeled with a scaffolding graph G = (V, E), where each node i ε V represents a contig and each edge (i, j) ε E represents all read pairs whose two individual reads are mapped to the contigs i and j, respectively. Each read in a pair is aligned either to the forward or reverse strand of corresponding contig sequence, and this results in 4 possible configurations for a read pair (denoted A, B, C, or D, see Figure S1 in Additional file 1) which can be modeled as a bidirected edge [23,30,33]. Orientation of contigs and the bidirected orientation of edges should agree (be concordant) with each other and should not result in any directed cycles for linear genomes (e.g. eukaryotes).

**Maximum likelihood scaffold graph orientation**. As an intermediate step towards solving the scaffolding problem, we consider the problem of determining an orientation of the scaffolding graph, which includes choosing one of the two possible orientations for each node (contig) i ε V as well as choosing for each edge (i, j) ε E one of the four bidirections that is concordant with the orientations of i and j. A common way to reduce an inference problem to an optimization problem is to seek a feasible solution with maximum likelihood. Let each observation, i.e., aligned read pair r ε R, have a probability pr of being correct. Any feasible contig orientation O = O(C) either agrees or disagrees with the read pair r. Let R_O _be the set of read pairs agreeing with O. Assuming independence of observations, the likelihood of an orientation O can be written as

$$\underset{r\in R_{O}}{\prod }p_{r}\underset{r\in R-R_{O}}{\prod }\left(1-p_{r}\right)=\underset{r\in R}{\prod }\left(1-p_{k}\right)\underset{r\in R_{O}}{\prod }\left(\frac{p_{r}}{1-p_{r}}\right)$$

and hence its log-likelihood is $\sum_{r\varepsilon R}\text{ln}\left(1-p_{r}\right)+\sum_{r\varepsilon R_{O}}\text{ln}\left(\frac{p_{r}}{1-p_{r}}\right)$. Since the first sum does not depend on the orientation O, maximizing the log-likelihood is equivalent to maximizing

(1) $$\underset{r\in R_{O}}{\sum }\text{ln}\left(\frac{p_{r}}{1-p_{r}}\right)$$

over all contig orientations O.

**Mapping probability estimation**. If p_r _'s are assumed to be the same for all read pairs, then the objective (1) reduces to maximization of the number of read pairs that agree with the contig orientation O. We consider the following factors that reduce the probability p_r _that read pair r is aligned correctly:

1. *Overlap with repeats*. As noted above, only pairs for which both reads map uniquely to the set of contigs are used for scaffolding. Still, a read that fully or partially overlaps a genomic repeat may be uniquely mapped to the incorrect location in case repeat copies are collapsed. We preprocess contigs to annotate repeats from known repeat families and by recording the location of multimapped reads. An estimate of the repeat-based mapping probability $p_{r}^{rep}$ is found by taking the percentage of bases of r aligned to non-repetitive portions of the contigs.

2. *Contig coverage dissimilarity*. Although sequencing coverage can have significant departures from uniformity due to biases introduced in library preparation and sequencing, the average coverage of adjacent contigs is expected to be similarly affected by such biases (all read alignments, including randomly allocated non-unique alignments, are used for estimating computing average contig coverages). If the two reads of r map to contigs i, respectively j, the coverage-based mapping probability of r, $p_{r}^{cov}$, is defined as 1 − |*coverage_i _− coverage_j _*|/(*coverage_i _+ coverage_j_*).

Note that factors such as repeat content of the sequenced genome and sequencing depth will determine how informative repeat-based and coverage-based mapping probabilities are. Depending on these factors, either $p_{r}^{rep}$, $p_{r}^{cov}$, or their product may provide the most accurate estimate for p_r_. Mismatches and indels in read alignments, that can be caused by sequencing errors or polymorphisms in the sequenced sample, can easily be incorporated in the estimation of mapping probabilities.

**Integer linear program**. Our integer linear program maximizes the log-likelihood of scaffold orientation using the following boolean variables:

- a binary variable S_i _for each contig i, with S_i _equal to 0 if the contig's orientation remains the same and S_i _= 1 if the contig's orientation is flipped w.r.t. default orientation in the final scaffold.

- a binary variable S_ij _for each edge (i, j) ε E, which equals 0 if none or both ith and jth contigs are flipped, and equals 1 if only one of them is flipped.

- binary variables A_ij _(respectively, B_ij _, C_ij _, and D_ij_) which are set to 1 if and only if an edge in state A (respectively, B, C, or D) is used to connect contigs i and j (see Figure S1 in Additional file 1). For any contig pair i and j, at most one of these variables can be one.

Let $A_{ij}^{r}$ (respectively, $B_{ij}^{r}$, $C_{ij}^{r}$ or $D_{ij}^{r}$) denote the set of read pairs supporting state A (respectively, B, C, or D), between the ith and jth contig. Define the constant $A_{ij}^{w}$ by

$$A_{ij}^{w}=\underset{r\in A_{ij}^{r}}{\sum }\text{ln}\left(\frac{p_{r}}{1-p_{r}}\right)$$

with $B_{ij}^{w}$, $C_{ij}^{w}$ and $D_{ij}^{w}$ defined analogously.

We now ready to formulate the ILP for maximizing the log-likelihood of a scaffold orientation:

(2) $$\underset{\left(i,j\right)\in E}{\sum }\left(A_{ij}^{w}\cdot A_{ij}+B_{ij}^{w}\cdot B_{ij}+C_{ij}^{w}\cdot C_{ij}+D_{ij}^{w}\cdot D_{ij}\right)$$

where

(3) $$S_{ij}\leq S_{i}+S_{j}\;S_{ij}\leq 2-S_{i}-S_{j}$$

(4) $$S_{ij}\geq S_{j}-S_{i}\;S_{ij}\geq S_{i}-S_{j}$$

(5) $$A_{ij}+D_{ij}\leq 1-S_{ij}\;B_{ij}+C_{ij}\leq S_{ij}$$

In this ILP, constraints (3-5) enforce agreement between contig orientation variables Si's and edge orientation variables S_ij_'s, A_ij_'s, B_ij_'s, C_ij_'s, and D_ij_'s.

Since eukaryotic genomes are linear, a valid scaffold orientation should not contain any cycles. The constraints (5) already forbid 2-cycles. Additionally, 3-cycles are forbidden with the constraints shown in Figure S2 in Additional file 1. Larger cycles generated in the ILP solution are broken heuristically because it is infeasible to forbid all of them using explicit constraints.

**Non-serial dynamic programming**. For large mammalian genomes, the number of variables and constraints is too large for solving the ILP (2)-(5) via standard solvers (SILP2 uses CPLEX [34] which is available free of charge for academic institutions). We adopt the non-serial dynamic programming (NSDP) paradigm to overcome this barrier and to optimally solve the problem. NSDP is based on the interaction graph with nodes corresponding to ILP variables and edges corresponding to the ILP constraints - two nodes are adjacent in the interaction graph if their associated variables appear together in the same constraint. Through the NSDP process, variables are removed in the way that adjacent vertices can be merged together [27]. The first step in NSDP is identifying weakly connected components of the interaction graph. We find the 2- and 3-connected components of the interaction graph with efficient algorithms and then we solve each component independently in such a way that the solutions can be merged together to find the global solution.

All constraints (3-5) as well 3-cycle constraints connect S_i_'s following the edges of the scaffolding graph. Therefore, the S_i_-nodes of the interaction graph for our ILP will have the same connectivity structure as the scaffolding graph G = (V, E). As it has been noticed in [23], the scaffolding graph is a bounded-width graph and should be well decomposable in 2- and 3-connected components. The SPQR-tree data structure is employed to determine the decomposition order for 3-connected components the scaffolding graph [28]. The solution to each component of the scaffolding graph is found using a bottom up traversal through which each component is solved 2 times: for similar and opposite orientations of the common nodes. The objective value of each case is then entered into the objective of the parental component. Having the solution of all components, top down DFS starting from the same root is performed to apply the chosen solution for each component.

**Figure 2:**
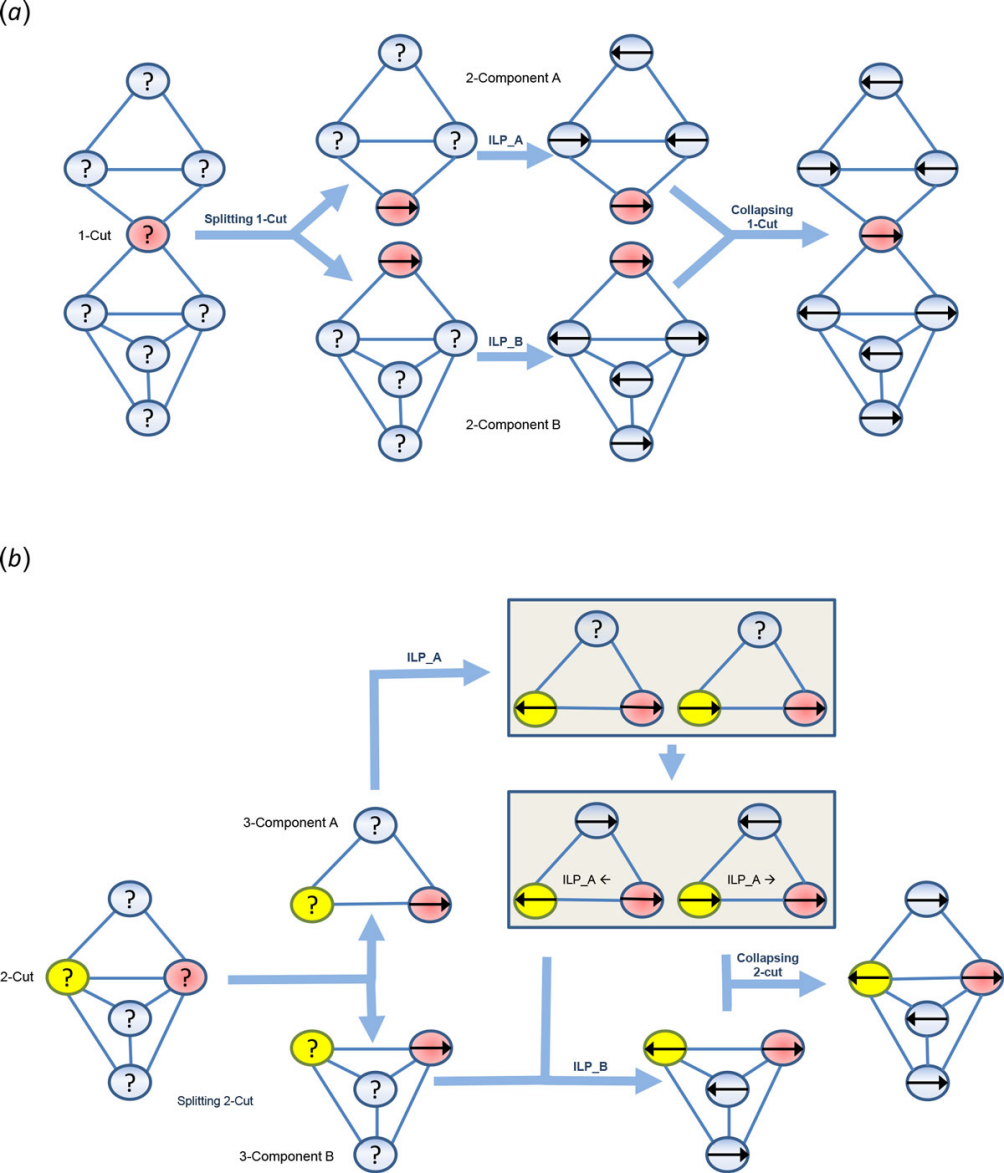
**Solving the maximum likelihood ILP via graph decomposition**. (*a*) Graph decomposition into 2-connected components: Red (1-cut) node splits the graph into two 2-connected components A and B. The ILP is solved for each component separately. If the direction of the cut node in the ILP solution for B is opposite to the one in the solution for A, then the solution of B is inverted. Then ILP solutions for A and B are collapsed into the parent solution. (*b*) Graph decomposition into two 3-connected components: Red and yellow (2-cut) nodes split the graph into two 3-connected components A and B. The ILP is solved for component A twice - for the same and the opposite directions assigned to two 2-cut nodes. Then these two solutions are used in the objective for the ILP of component B. Finally, ILP solutions for A and B are collapsed into the parent solution.

Below we illustrate the way how the solution is computed in stages through each of which the results of the previous stage are combined to dynamically solve the problem. Obviously, an isolated connected component will not influence other components. Moreover, it has been shown in [21] that 2-connected components can be solved independently. As it can be seen in Figure 2(a), after removing the articulation point (1-cut) to decompose the graph into 2-connected components, each component is solved with the same arbitrary direction assigned to the common node, and then the resulting solutions are collapsed into the parent solution. The pre-assigned direction will never affect the parent solution since all contigs in the scaffold can be flipped at the same time.

Still, 2-connected components can be very large, so we look for 2-cuts in order to decompose the graph into significantly smaller 3-connected components. Figure 2(b) shows that splitting the two 2-cut nodes i and j decomposes the graph into 3-connected components A and B. The ILP for component A is solved twice to obtain

(1) the ILP solution sol_00 _in which the 2-cut nodes i and j are constrained to both have default orientations;

(2) the ILP solution sol_01 _in which the 2-cut nodes i and j are constrained to have opposite orientations.

The two solutions are combined to solve the ILP for component B. The ILP objective for component B should be updated by adding the term of sol_00_+(sol_01_-sol_00_) · S_ij _or, equivalently, the value sol_00 _should be added to $A_{ij}^{w}$ and $D_{ij}^{w}$ and the value sol_01 _should be added to $B_{ij}^{w}$ and $C_{ij}^{w}$. The overall solution is obtained by identifying the common nodes of the components. In the example on Figure 2(b), the optimal solution happens when 2-cut nodes have opposite directions. The corresponding solution of ILP for the component A should be incorporated in the overall solution. When the scaffolding graph has 3-connected components too large to handle, 3-cuts could also be used for decomposition.

**Figure 3:**
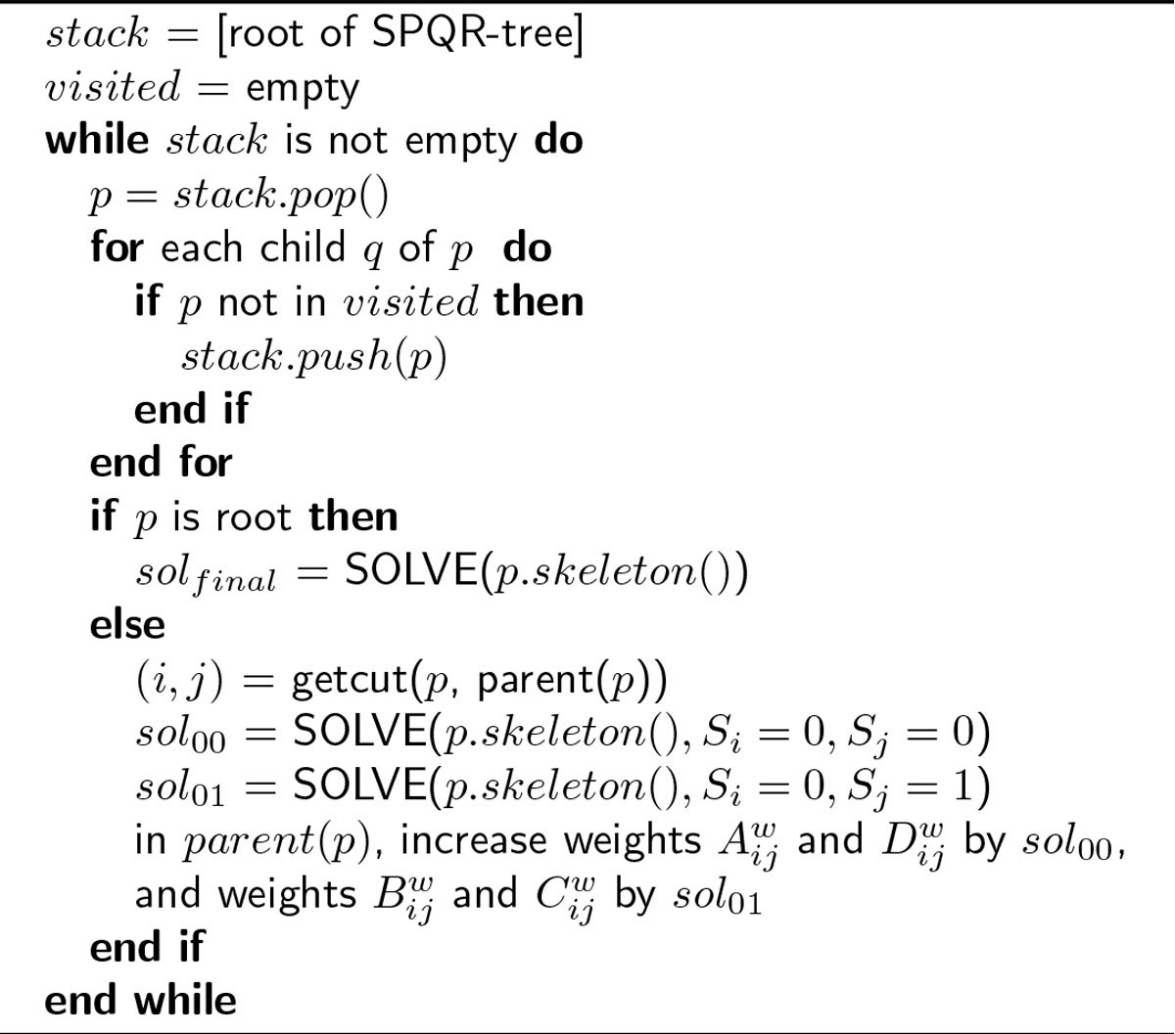
**SILP2's NSDP algorithm for processing 3-connected components**.

The pseudo-code of the SILP2 NSDP algorithm for processing 3-connected components is given in Figure 3 SILP2 is different from SILP1 in the else clause - instead of solving ILP for each of four possible combinations of assignments for S_i _and S_j _as in SILP1, ILP is solved only two times for combinations S_i _= 0 & S_j _= 0 and S_i _= 0 & S_j _= 1.

**Thinning heuristic**. Unfortunately the largest tri-connected component may still induce an ILP too large for CPLEX to solve in a reasonable amount of time. In order to address this problem a thinning heuristic is applied to the scaffolding graph. This scenario can be detected by setting a threshold on the maximum number of contigs allowed in a tri-connected component. When a component exceeds the threshold the number of read pairs necessary to induce an edge is increased by one and decomposition recomputed until there is no component above the threshold.

## Results and discussion

### Datasets and quality measures

In order to asses the quality and scalability of our scaffolding tool we developed a testing framework which closely mimics real world scaffolding problems. We utilized the Staphylococcus aureus (staph), Rhodobacter sphaeroides (rhodo) genomes and chromosome 14 of HapMap individual NA12878 (chr14) from the GAGE [14] assembly comparison. Finally, in a test case designed to stress scalability, contigs from a draft assembly of individual NA12878 (NA) created by [35] were scaffolded using short-read data.

In all test cases the read pairs used for scaffolding are aligned against the contigs using bowtie2 [36]. Each read in a pair was required to be aligned uniquely according to the default scoring scheme, for the pair to be considered valid. Each scaffolder was given the same set of valid read pairs. Two of the leading external scaffolding tools MIP [21] and OPERA [37] are used in this comparison. Although many other tools do exist, these two are widely utilized and actively maintained.

The three small test cases are used to test both correctness and scalability of the scaffolding tool. In order to test correctness, contigs simulating a draft assembly were created by placing gaps in the genome. The contig and gap sizes were sampled uniformly at random from the collection of all the assemblies used in the GAGE comparison. The procedure to generate the contigs was to alternatively sample with replacement from the set of all contig sizes, and gap sizes. In this way a simulated scaffold can be generated so that the position and relative orientation of all contigs and all gap sizes are known. The orientation of the simulated contigs was randomized to prevent biases.

For each genome 10 replicates were created, all subsequent results are the average of the 10 replicates. By creating simulated contigs with no assembly error, the accuracy of subsequent scaffolds can be evaluated exactly. Although the contigs were simulated, real read pairs were aligned against them and used as input. Table S1 in Additional File 1 describes the characteristics of each dataset.

The NA12878 test case was produced by simply using the contigs created in the SGA [35] assembler publication. The read pairs were obtained from a different lab, however they were generated using the same biological source material (ERP002490). Although more read pairs were available a random subset of approximately 2x coverage was used.

Finally a simulated metagenomics test case was created to explore the feasibility of utilizing SILP2 to scaffold metagenomes. This was created by artificially mixing the staph and rhodo contigs and reads at varying proportions.

A natural and common parameter present in all scaffolding algorithms in the bundle size, or the number of read pairs spanning two contigs. This parameter is a natural control of sensitivity and specificity; requiring more support increases specificity at the price of sensitivity and vice-versa. It should be noted that every scaffolding tool tested, including SILP2 does not abide by the set parameter absolutely. Each method raises it in order to ensure efficient operation. The simulated test cases were evaluated at several bundle sizes to asses its effect on accuracy and scalability. The NA12878 test case was only evaluated at the minimum feasible value due to resource constraints.

## Accuracy

Calculating the accuracy of de novo assemblies or scaffolds is quite difficult. One of the key challenges is deciding on the appropriate measure. In this comparison we elect to present several metrics which will likely have different weight depending on the background and intention of the reader.

For the simulated contigs we treat scaffolding as a binary classification problem where methods attempt to predict true adjacencies in the test dataset. The accuracy and sensitivity can be directly measured by computing true positive, and false positive rates. One common summary is MCC, or Mathews Correlation coefficient. This measure assess sensitivity and specificity simultaneously. In the context of scaffolding, this measure illustrates how many correctly ordered and oriented scaffolds were created.

An alternative measure, commonly utilized in genome assembly comparison publications [14,38] is the notion of corrected N50. Where N50 is the weighted mean scaffold size, the corrected N50 is the same statistic after errors are removed. This can be computed exactly on simulated data, however an alignment based approximation must be used on real test cases.

Finally the usefulness of a genome can also be measured by the number of identifiable biological features captured. Here we capture this measure by recording the percentage of known genes that are found contiguous in the scaffolds.

### MCC

**Figure 4:**
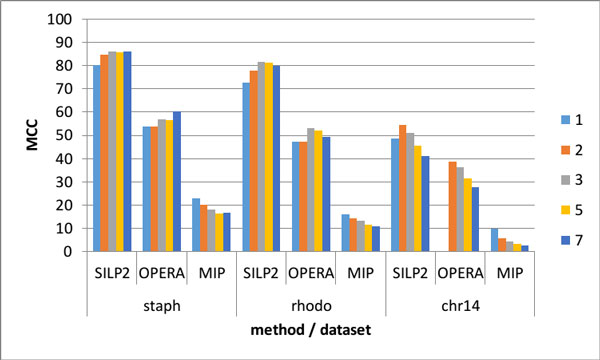
**MCC comparison**. MCC of SILP2, OPERA, and MIP across bundle sizes for staph, rhodo, and chr14 datasets. At bundle size 1 OPERA exceeded the allowed runtime of 2 days for the chr14 dataset.

The MCC metric, as seen in Figure 4, indicates that SILP2 is able to correctly join the most contigs, followed by OPERA and finally MIP. This order holds for all three simulated test cases. Interestingly all three methods see a decrease in MCC on staph, but some have increases on rhodo and chr14. This trend illustrates the difficult to define variables such as genome uniqueness, assembly and read error which can make smaller genomes more challenging that larger genomes.

While MCC is natural to a computer scientist its useful to a biologist is lacking because the content of the contigs is ignored. A biologist typically asses a scaffold by the N50, Unfortunately this measure does not reflect the accuracy of the scaffolds and rewards aggressive merging. Using MCC or its constituent components as metrics gives greater clarity to the researcher comparing different tools.

### N50

The most common metric found in genome assembly and scaffolding is N50. The most recent iteration of benchmark projects have transformed this descriptive number into an accuracy measure by introducing alignment based corrections. Here the scaffolds are aligned against a reference and miss-alignments are interpreted as orientation, or placement errors. We have developed a more efficient implementation of the correcting method developed by [38]. This enables the tool to be utilized on the NA12878 test case at the cost of accuracy.

The true N50 value can be determined when using simulated contigs by breaking incorrect scaffolds, this measure is denoted as TPN50. An analog to the TPN50 measure can be obtained by aligning the scaffolds against the known reference. Scaffolds (and contigs) are broken at mis-assembled or mis-scaffolded regions. This post-alignment metrics can be obtained from the assembly evaluation tool called QUAST [38] and it is denoted as NA50.

**Figure 5:**
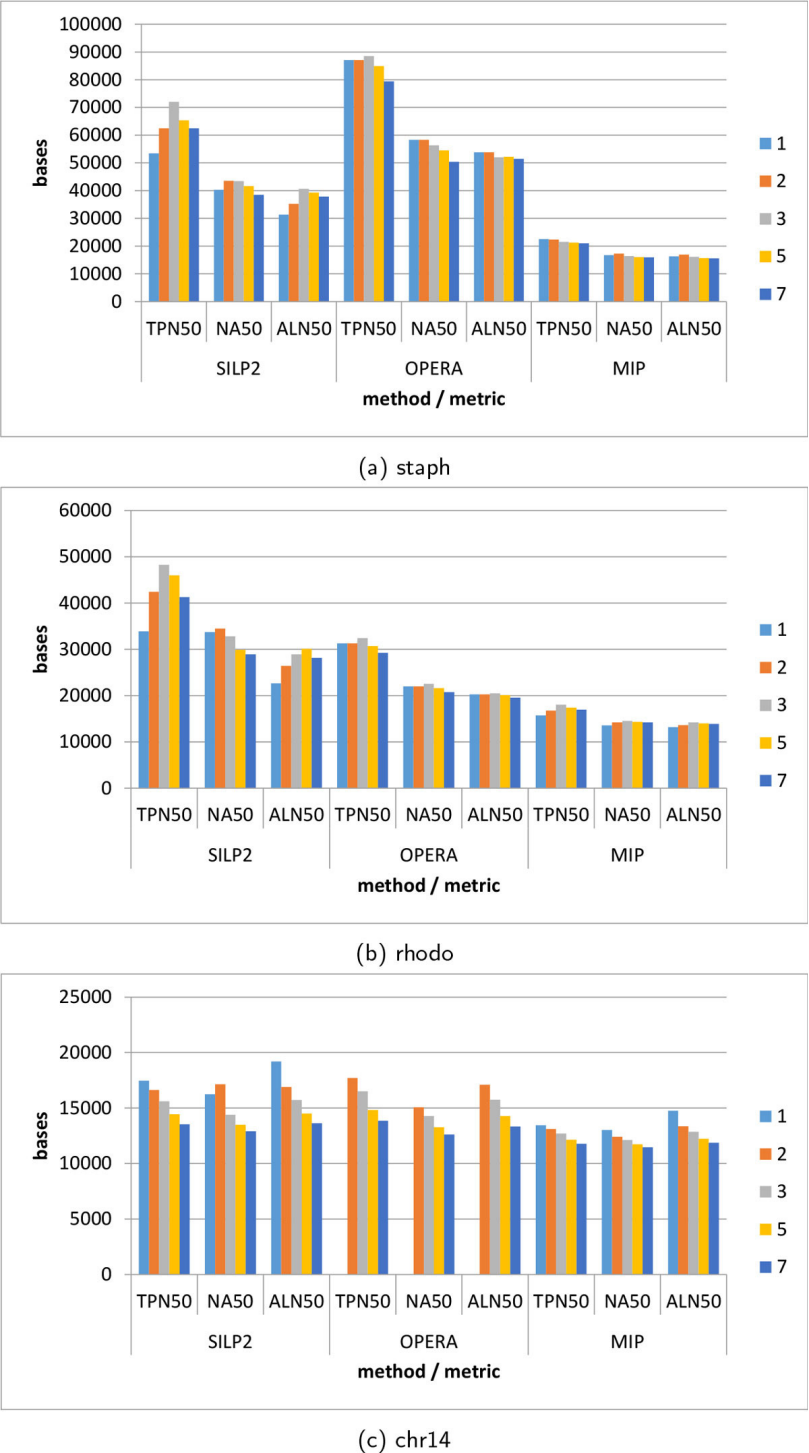
**TPN50, ALN50 and NA50 as a function of bundle size for single-genome datasets**. TPN50 is obtained by breaking incorrect scaffolds, ALN50 is the post-alignment metric developed by us, and NA50 is the QUAST equivalent. The colors indicated in the legend correspond to the bundle size 1 through 7. OPERA was unable to complete on bundle size 1 for chr14 dataset.

Unfortunately the implementation of QUAST required more than 128GB of RAM to evaluate the NA12878 test case, and therefore could not be run. We wrote an alternative implementation of NA50 called ALN50 which is more efficient, but follows a similar framework. Both NA50 and ALN50 are found in Figure 5. Although NA50 and ALN50 do not agree, they do indicate similar trends between methods. Therefore ALN50 will be used henceforth. In the staph genome, OPERA is clearly the best performing tool, followed by SILP2 and then MIP. However on the rhodo genome, SILP2 performs best, followed by OPERA then MIP.

First the highest ALN50 is always found at bundle size 3 or 5. If the intent of the assembly is to maximize N50 then clearly no algorithm should be run with bundle size less than 3. However, as it was pointed out in both GAGE and QUAST [14,38], N50 is a misleading metric and alternative measures may be a better judge.

Additionally it can be seen that both OPERA and SILP2 have approximately the same TPN50 in the staph and chr14 test cases, however in rhodo, SILP2 clearly out-performs OPERA and MIP at all bundle sizes. It is not clear why SILP2 performs much better on rhodo, and approximately equivalent on the others. For the complete genome SILP2, OPERA and MIP reported an N50 of 26,235, 39,366, 26,235 respectively. This is consistent with the observations from the synthetic data sets.

### Gene reconstruction

An alternative measure of the completeness of a scaffold is the number of genes aligned against the scaffold. For a given percentage of completeness the number of genes found in the corrected scaffold is an indicator of the usefulness of the genome.

As seen in Table 1, SILP2 almost consistently equals or outperforms both OPERA and MIP at all bundle sizes and for each genome. The difference between SILP2 and MIP is often quite small.

**Table 1:** The number of reconstructed genes found in the corrected scaffolds for single-genome datasets.

genome	bundle	SILP2	OPERA	MIP	total
staph	1	1,727.70	1,168.50	1,545.00	2692
	2	1,727.70	1,168.50	1,559.50	
	3	1,727.70	1,210.60	1,575.30	
	5	1,727.70	1,262.70	1,584.60	
	7	1,727.40	1,280.40	1,588.50	

rhodo	1	2022.7	1618.6	1897.3	3067
	2	2022.7	1618.6	1907	
	3	2022.6	1751.1	1894	
	5	2022.6	1834.2	1921.3	
	7	2022.6	1853.3	1933.3	

chr14	1	350.9	-	349.6	529
	2	352.00	330.10	350.40	
	3	352.40	336.90	350.40	
	5	352.40	337.50	351.70	
	7	352.40	337.60	3.00	

NA12878 2x	1	30817	-	30817	34039
	2	30850	30809	30849	

In order for a gene to be considered reconstructed 90% of its sequence must be found in a contiguous scaffold. Dashes (-) indicate the method was unable to complete and therefore the gene count could not be computed.

### Runtime

One key advantage of SILP2 over other scaffolding tools is its speed and scalability. Table 2 gives the runtime of SILP1, SILP2, OPERA and MIP on single-genome testcases. All experiments were conducted on a Dell PowerEdge R815 server with quad 2.5GHz 16-core AMD Opteron 6380 processors and 256Gb RAM running under Ubuntu 12.04 LTS. IBM ILOG CPLEX 12.5.0.0 was used as ILP solver through the CPLEX Python API. Reported runtimes are only for the scaffolding portion of each program. Read alignment and pre-processing steps are not included, but it was observed that all methods had comparable pre-processing times.

**Table 2:** Runtime (in seconds) for scaffolding single-genome datasets.

genome	bundle	SILP1	SILP2	OPERA	MIP
staph	1	1237	6.4	2538.1	35.8
	2	738	4.5	1456.5	17
	3	305	4	878.5	12.834
	5	142	3.9	386.9	10.54
	7	51	4.3	241	10.115

rhodo	1	1134	10	2297	118.953
	2	632	4.1	455.2	25.3
	3	486	3.6	5.7	10.995
	5	86	3.4	2	8.778
	7	75	3	1.6	8.217

chr14	1	-	64.7	-	706.3
	2	-	27.6	99.25	189.685
	3	629	25.5	11	137.67
	5	370	21.5	12	107.85
	7	400	19.25	10.75	94.9875

NA12878 2x	1	-	55.2	-	89.3
	2	-	1670	76.49	53.28
	3	37751	3878	7875	121.61
	5	27341	3183	4270	134.6
	7	27470	3626	2180	125.66

All timing was captured only during the scaffolding phase of each tool, all read alignment and formatting procedures were excluded from timing. The number is the average of 10 runs for each genome. A dash (-) indicated the tool was unable to complete in the allotted time of 2 days for staph,rhodo,chr14 and 3 days for NA12878.

On the staph, rhodo and chr14 datasets, it was observed that SILP2 was quicker at higher bundle sizes and no worse than OPERA or MIP at lower bundle sizes. The NA12878 testcase was extremely challenging for all methods and demonstrated the effect of heuristics on large test cases. It is clear from the reduced runtimes that all 3 methods activate some sort of heuristic at lower bundle sizes. The difference between SILP1 and SILP2 is evident at all bundle sizes.

The NA12878 genome was also scaffolded by SILP2 using 20x coverage reads, with a runtime of 18,205 seconds at bundle size 1. Negligible improvement in accuracy over the 2x dataset was observed. From Table 2 it is clear that runtime increases with the complexity of the genome more so than the number of read pairs.

## Metagenomics

Metagenomics is the study of genetic material recovered from heterogeneous mixtures often found in nature. Just like in the de novo assembly of a single genome, the accuracy and size of the scaffolds is critical to subsequent analysis steps. Our ILP based solution is flexible enough to include new constraints and objectives to better serve this challenging scenario.

In order to test this hypothesis a simulated metagenomic dataset was created utilizing the staph and rhodo genomes from the GAGE dataset. The simulated contigs used previously were mixed, and both sets of reads were aligned with varying fractions (1.0, 9.5 0.25, 0.0) of staph reads present. Again all three of the major scaffolding tools were tested, however additional weighting scenarios were implemented in SILP2.

The runtime, MCC, SCFN50, TPN50 and ALN50 metrics are detailed for each of the compared methods in Table 3. Also an additional scaffolding tool BAMBUS2 [39] was added to the comparison because it was previously shown to work well in the metagenomic scaffolding context.

**Table 3:** Combined runtime and accuracy results for the simulated low-complexity metagenome datasets.

METHOD	FRAC STAPH	RUNTIME	SCFN50	ALN50	TPN50	MCC
SILP2 0	1.00	14.3	51,775.0	20,647	34,495	67.6
SILP2 0	0.50	13.3	50,450.0	20,103	36,356	69.1
SILP2 0	0.25	12.7	47,731.0	20,761	35,323	69.3
SILP2 0	0.00	11.5	21,753.0	13,948	15,649	39.9

SILP2 1	1.00	14.3	52,557.0	20,655	33,683	67.5
SILP2 1	0.50	13.8	48,701.0	20,337	35,750	69.0
SILP2 1	0.25	13.4	49,766.0	20,752	35,146	69.0
SILP2 1	0.00	11.0	21,925.0	13,847	15,511	39.3

SILP2 2	1.00	14.3	43,144.0	20,631	31,160	66.3
SILP2 2	0.50	13.5	42,244.0	20,198	32,477	67.5
SILP2 2	0.25	13.2	45,137.0	21,161	31,562	67.6
SILP2 2	0.00	10.8	22,190.0	13,813	16,205	41.6

SILP2 3	1.00	14.1	43,646.0	19,998	28,856	65.3
SILP2 3	0.50	13.2	41,893.0	19,790	30,504	66.6
SILP2 3	0.25	13.0	42,188.0	19,945	30,449	66.4
SILP2 3	0.00	11.5	21,820.0	13,781	15,635	40.0

OPERA	1.00	2247.2	15,573.0	13,082	10,386	10.1
OPERA	0.50	1567.6	13,928.0	12,006	10,440	10.7
OPERA	0.25	884.0	14,786.0	12,617	10,507	10.5
OPERA	0.00	544.3	11,121.0	10,720	10,273	4.9

MIP	1.00	129.9	20,104.0	12,861	18,672	18.4
MIP	0.50	121.3	19,807.0	12,488	17,613	17.4
MIP	0.25	114.0	18,520.0	12,269	16,680	17.2
MIP	0.00	114.1	12,690.0	10,894	12,434	8.7

BAMBUS2	1.00	1025.89	11,251.0	11,238	-	-
BAMBUS2	0.50	1452.75	10,781.0	10,822	-	-
BAMBUS2	0.25	1676.75	10,806.0	10,834	-	-
BAMBUS2	0.00	2272	11,526.0	11,698	-	-

The second column indicates the percentage of total read pairs used from the staph genome testcase, all of the rhodo pairs were used. SCFN50 is the uncorrected N50 reported by each scaffolding tool. The N50 of the contigs alone is 10,339 bp. The integer appended to SILP2 indicates the bundle weight; 0: none, 1: coverage, 2: repeat, 3:coverage * repeat. All methods were run at bundle size 1, the reported number is the average of 10 runs except for OPERA where 6 of the test cases exceeded runtime limits. TPN50 and MCC were unable to be computed for BAMBUS2 because the generated AGP had a non-standard format.

Interestingly all SILP2 variants fare much better than both OPERA, MIP and BAMBUS2 even with no staph reads present (this differs from results in Figure 5 because the rhodo reads were aligned to both staph and rhodo contigs). It is unclear is the different methodology used in SILP2 sets it apart, or if an implementation quirk throws off the other scaffolders. However across all metrics SILP2 variants perform the best.

In both SILP2 variants and MIP it is observed that the TPN50 decreases as fewer staph reads are utilized. This is expected since there are fewer opportunities to connect staph contigs and both staph and rhodo contigs are used in the calculation of N50. There is no major differences between the variants of SILP2. The coverage based weight seems to improve MCC at the cost of a slightly lowered TPN50 when compared to no weights.

This highly simplified test scenario is not designed to fully explore metagenomic scaffolding, rather to point out an opportunity to further external genome scaffolding algorithms.

## Conclusions

Scaffolding in an important step in the de novo assembly pipeline. Biologists rely on an accurate scaffold to perform many types of analysis. The larger the scaffold the more useful it will be to them. Recent advances in de novo assemblers has made it feasible to create draft assemblies for large mammalian genomes. We believe the SILP2, coupled with the most recent scalable assemblers will produce the largest and most complete assemblies. This is made possible utilizing non-serial dynamic programming approach to solve our robust ILP. The ILP formulation for the maximum likelihood model is shown to be flexible enough to handle metagenomic samples.

The future work includes more thorough experimental validation of SILP2 and comparison BAMBUS2 [39] on metagenomic samples. Also we are going to validate SILP2 using methodology and benchmarks from recent paper [40].

## List of abbreviations

• ILP - Integer Linear Programming

• MCC - Matthew's correlation coefficient

• MIP - Mixed Integer Programming

• NSDP - Non-serial Dynamic Programming

• OGDF - Open Graph Drawing Framework

• SQPR - a tree data structure used in graph algorithms to represent the 3-connected components of a graph [29]

## Software and data availability

A reference implementation of SILP2 is provided at https://github.com/jim-bo/silp2. This implementation relies on the CPLEX optimization library. The code used for generating most of the reported results, including the implementation of the ALN50 metric, is available at https://github.com/jim-bo/scafathon. The SILP2 source code along with a small test dataset can also be downloaded from http://dna.engr.uconn.edu/software/SILP2.

## Authors' contributions

JL developed and implemented the algorithms, ran the experiments, and wrote result section, HS developed algorithms, participate in writing the paper, and implemented parts of the code, IM and AZ developed algorithms wrote the paper, conceived and supervised implementation.

## Supplementary Material

Additional file 1 **Supplementary figures, methods, and tables are supplied in PDF format**. Click here for file

## Declarations

The authors declare that funding for publication of the article was by the research institute of the corresponding author.

This article has been published as part of BMC Bioinformatics Volume 15 Supplement 9, 2014: Proceedings of the Fourth Annual RECOMB Satellite Workshop on Massively Parallel Sequencing (RECOMB-Seq 2014). The full contents of the supplement are available online at http://www.biomedcentral.com/bmcbioinformatics/supplements/15/S9.
