# Supplementary material for: ILP-based maximum likelihood genome scaffolding
Source: BMC Bioinformatics. 2014 Sep 10;15(Suppl 9):S9. doi: 10.1186/1471-2105-15-S9-S9 (PMC4168704; doi:10.1186/1471-2105-15-S9-S9)
Supplement: Additional file 1 — Supplementary figures, methods, and tables are supplied in PDF format. [file 1471-2105-15-S9-S9-S1.pdf]

Supplementary figures, methods, and tables

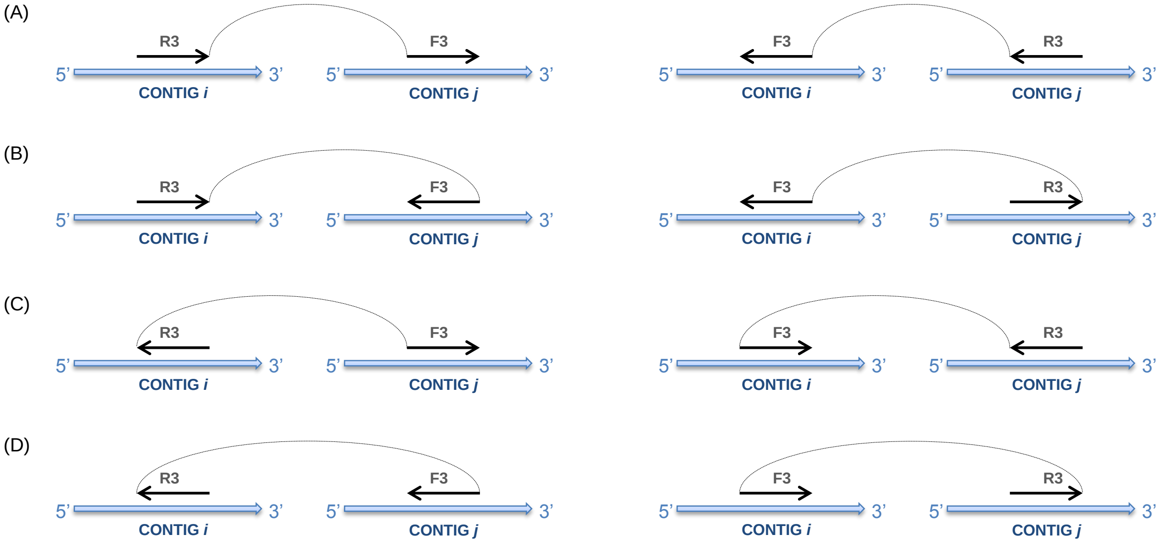

Figure S1: The four possible orientations of a read mate-pair linking two contigs i and j.

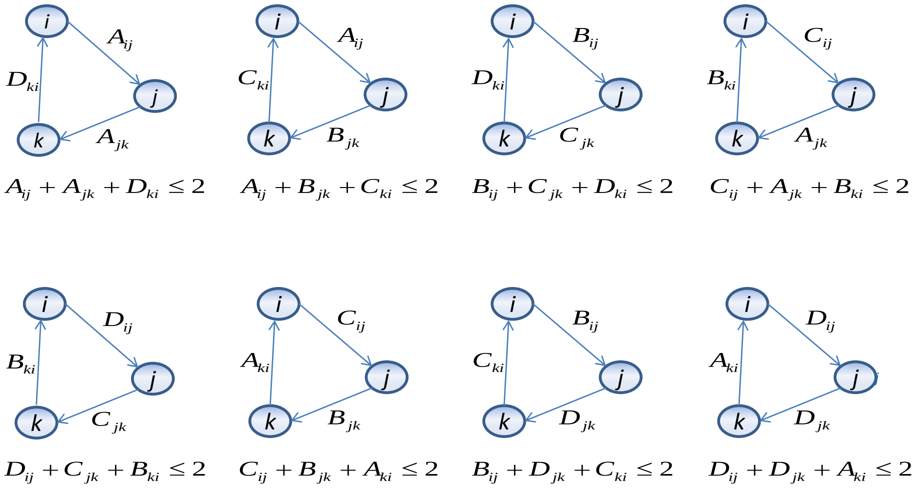

Figure S2: ILP constraints forbiding 3-cycles.

S1 Contig ordering via bipartite matching

The solution to the ILP does not yield a scaffold, instead only gives the pairwise order and orientation of the contigs (see Figure S3(a)). In order to find scaffolding, we need remove edges such that remaining graph becomes a collection of paths. Naturally, we want to remove the least probable edges, i.e., lightest edges. This problem can be solved efficiently by finding matching in the following bipartite graph $B = (V^1 \cup V^2, E)$ where each vertex in V^1 corresponds to the 3' end of a contig, each vertex in V^2 to the 5' end of a contig, and each edge corresponds to an edge from ILP solution connecting the 5' of its beginning to the 3' end of its end (see Figure S3(b)). Each edge in B has the same weight as in the ILP solution. Obviously, any matching in B corresponds to a collection of paths and simple directed cycles (see Figure S3(c)). Then from each cycle the lightest is deleted and we output the resulted set of paths.

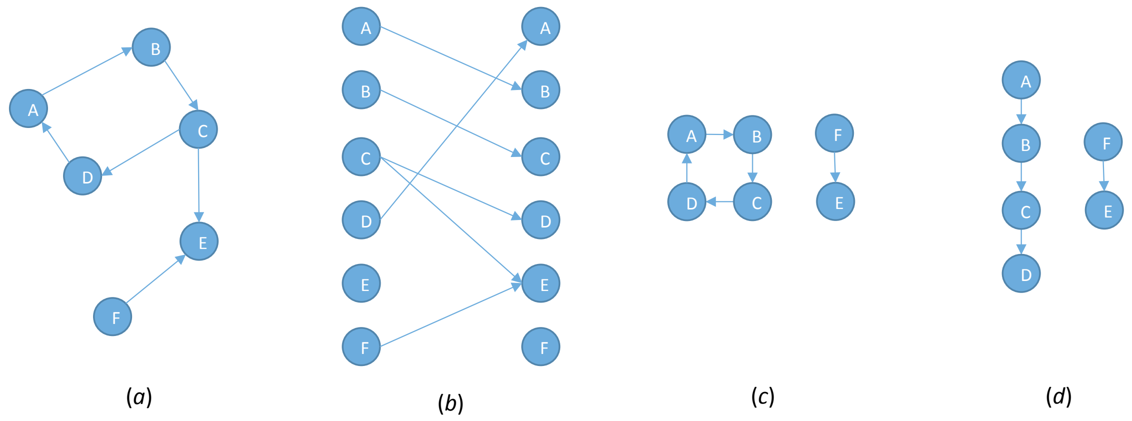

Figure S3: ***(a)*** Pairwise ordering obtained from ILP output. ***(b)*** The corresponding bipartite graph. ***(c)*** The collection of simple paths and cycles in the bipartite matching. ***(d)*** The scaffold represented by a collection of paths obtained by deletion of the lightest edges from simple cycles.

S2 Maximum likelihood gap-length estimation

Having the scaffold chains, gap sizes between consecutive contigs are estimated using the constraints imposed by the paired-reads. This is the final step of scaffolding towards building finished genomes. As shown in Figure S4, paired reads might spread over more than one gap in the scaffold path (green and red edges). The maximum number of gaps spanned by a read highly depends on the read library inset size and the minimum contig length. On the other hand, a single gap can be jumped over by multiple reads. Each read indicates a total length for gaps expanded. Since different reads could indicate different lengths for the same gap, we adopt the maximum likelihood (ML) approach provided in OPERA [1] to compute the gap sizes.

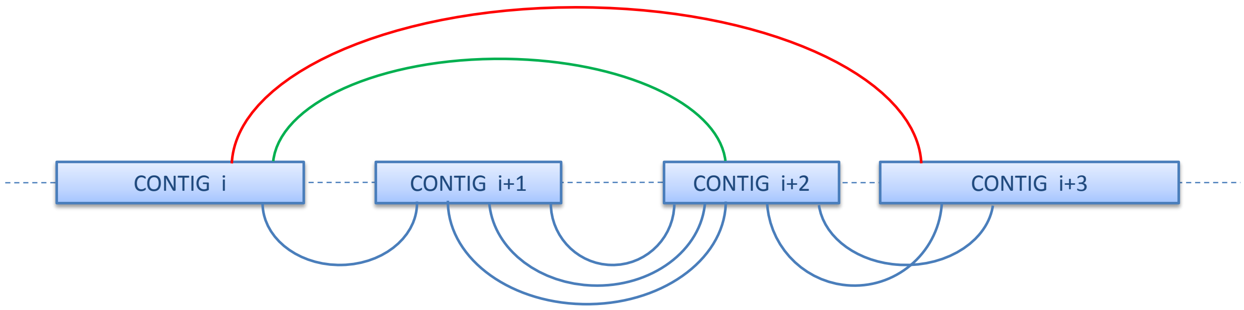

Figure S4: Paired reads connecting contigs in a scaffold chain: Blue reads span only one gap while the green and red reads span two and three gaps, respectively.

Table S1: Summary statistics for the single-genome datasets used in the empirical evaluation. Staph, rhodo and chr14 contigs were simulated by randomly selecting from the finished genome sequence contigs with length distribution mathcing the length distribution of contigs assembled in [2]. The NA12878 contigs were generated using the SGA assembler in [3].

Dataset	Genome size	# contigs	Pair count	Read size	Avg. insert size	Read cov.	Physical cov.
staph	2.9 MB	335	1.7 M	37 bp	3500 bp	43 X	2052 X
rhodo	4.6 MB	369	1 M	100 bp	3500 bp	43 X	761 X
chr14	107 MB	10355	11 M	100 bp	2500 bp	21 X	257 X
NA12878 2x	3.0 GB	583982	25 M	100 bp	2500 bp	2 X	21 X
NA12878 20x	3.0 GB	583982	160 M	100 bp	2500 bp	11 X	133 X

References

1. Gao S, Nagarajan N, Sung WK: **Opera: reconstructing optimal genomic scaffolds with high-throughput paired-end sequences**. In *Proc. 15th Annual international conference on Research in computational molecular biology* 2011:437–451.
2. Salzberg SL, Phillippy AM, Zimin A, Puiu D, Magoc T, Koren S, Treangen TJ, Schatz MC, Delcher AL, Roberts M, Marçais G, Pop M, Yorke JA: **GAGE: A critical evaluation of genome assemblies and assembly algorithms**. *Genome Research* 2012, **22**(3):557–567, [http://genome.cshlp.org/content/22/3/557.abstract].
3. Simpson JT, Durbin R: **Efficient de novo assembly of large genomes using compressed data structures**. *Genome Research* 2012, **22**(3):549–556, [http://genome.cshlp.org/content/22/3/549.abstract].
